# Renal Cell Carcinoma Metastasis to the Small Bowel: A Rare Finding 10 Years After Nephrectomy

**DOI:** 10.7759/cureus.53141

**Published:** 2024-01-29

**Authors:** David Toro Tole, Jonathon Bowden, Sheshang Kamath, Manmeet Kaur, Domenic LaPaglia

**Affiliations:** 1 Surgery, Royal Darwin Hospital, Darwin, AUS; 2 Surgery, Flinders University, Darwin, AUS

**Keywords:** case report, metastasis, ileum, small bowel, renal cell carcinoma

## Abstract

Renal cell carcinoma (RCC) most commonly metastasizes to the lung, lymph nodes, bone, and liver. RCC metastasizing to the small bowel is exceedingly rare (0.7%), and the ileum is the least likely site. We discuss the case of a 63-year-old male patient who presented with melena and a 10-kg unintentional weight loss in the preceding month 10 years after undergoing curative nephrectomy for RCC; he was found to have an ileal mass on CT imaging. He subsequently underwent a diagnostic gastroscopy and laparoscopy, later converted to a laparotomy, with bowel resection and anastomosis. The immunohistochemistry was consistent with RCC. This case report highlights the need for awareness about this rare but potential site of metastasis, which may present with gastrointestinal bleeding.

## Introduction

Renal cell carcinoma (RCC) accounts for approximately 90% of all primary renal neoplasms. These predominantly include clear cell RCC (70%), papillary RCC (10-15%), and chromophobe RCC (5%) [1,2]. Males are more commonly affected than females, and most cases are observed in those aged between 50 and 70 years [3,4]. At the time of presentation, 30% of individuals will have metastasis of their primary tumor [5]. The most common sites of metastasis are the lungs, followed by bone, lymph nodes, liver, and brain, while the involvement of the small bowel is exceedingly rare [6]. Furthermore, 20-40% of patients with metastatic renal carcinoma (mRCC) will also develop metastatic disease recurrence [7]. The likelihood of survival is closely tied to the stage of diagnosis. While stage I is associated with a five-year relative survival rate of 93%, it is 72.5% for stage II/III. However, for stage IV metastatic disease, the survival rate drops significantly to just 12% [1].

Metastasis to the small bowel has been reported in as low as 0.7% of patients [1]. The most common presenting clinical symptoms of metastasis to the small bowel from RCC are melena or haematochezia, abdominal pain, or systemic features such as weight loss. Metastasis from RCC has been found to occur in patients years after undergoing radical nephrectomy. Clear cell RCC has been found to be the predominant type of RCC to cause these types of metastases. Jejunal and duodenal metastases are found to be more common than ileal metastases [7].

Metachronous metastasis is defined as metastasis occurring >6 months after partial or radical nephrectomy for localized RCC and is considered to be rare, as reflected by scarce literature on the subject. Independent studies have shown that 81.8% of patients who present with metachronous mRCC are older than 60 years [7]. We discuss a rare case of RCC metastasis to the small bowel. This report explores the presentation and diagnosis of a male patient who presented 10 years post radical nephrectomy with a metachronous metastasis of RCC to his ileum.

## Case presentation

A 63-year-old male patient presented to the Emergency Department with a three-week history of melena and a 10-kg unintentional weight loss in the preceding month. He also reported lethargy and dizziness. He had a history of a right laparoscopic nephrectomy 10 years prior for a lesion initially presumed benign, with no associated histopathological reports available. He had not undergone any previous colonoscopies or gastroscopies. He had no known family history of malignancy.

On examination, he was hemodynamically stable, pale, with a soft non-tender abdomen, and without palpable masses. His full blood count showed features of iron deficiency anemia and reactive thrombocytopenia. All other serological investigations were within normal limits. A CT of the abdomen and pelvis showed a 4.6 x 4.0 x 3.8 cm mid-ileum irregular mass with intra- and extra-luminal extension, associated with vessels extending into the mass from the mesentery, and a 0.9 cm mesenteric node. He proceeded to have a staging CT of the chest and brain, which showed no evidence of metastatic disease. No features of concern were identified in the contralateral kidney. He underwent an esophagogastroduodenoscopy (EGD) of the duodenum, which was unremarkable. He also had a staging laparoscopy, which identified a mid-ileal fungating mass. The procedure was then converted to a laparotomy, and a 14 cm section of bowel was resected with an end-to-end anastomosis. No peritoneal disease was identified.

**Figure 1:**
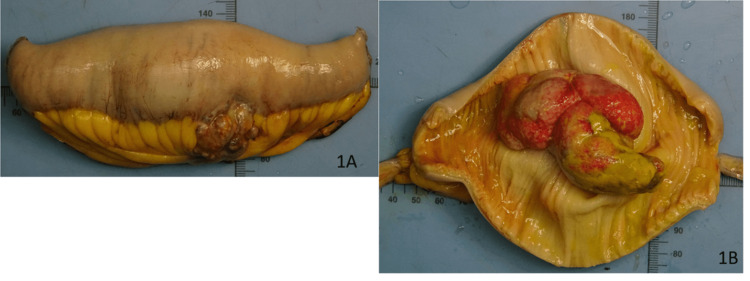
Macroscopic specimen of the resected small bowel 1A: tumor invading serosa and mesentery; 1B: polypoid tumor protruding in the lumen with ulceration of overlying mucosa

**Figure 2:**
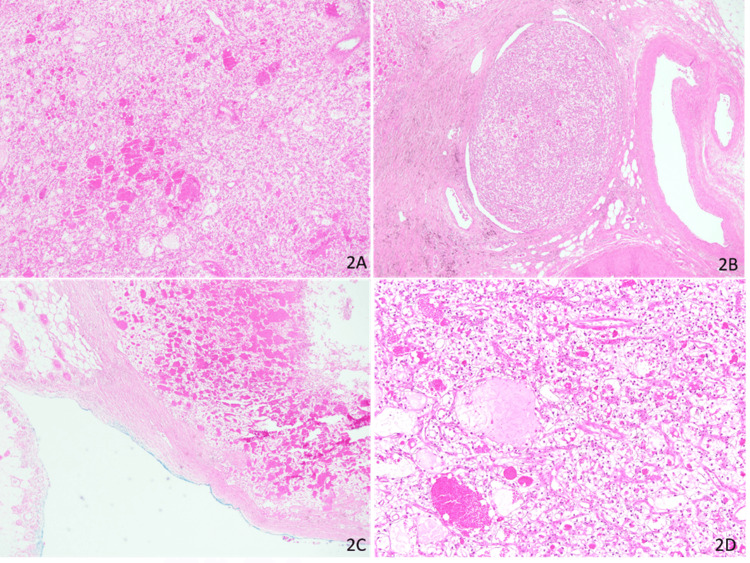
Microscopic specimen, H&E stain 2A: tumor arranged in sheets with microcystic areas and delicate vasculature; 2B: vascular tumor emboli seen; 2C: tumor involving serosa and mesentery; 2D: tumor cells with clear cytoplasm and borders (2A, B, and C: 10X magnification. 2D: 40X magnification)

**Figure 3:**
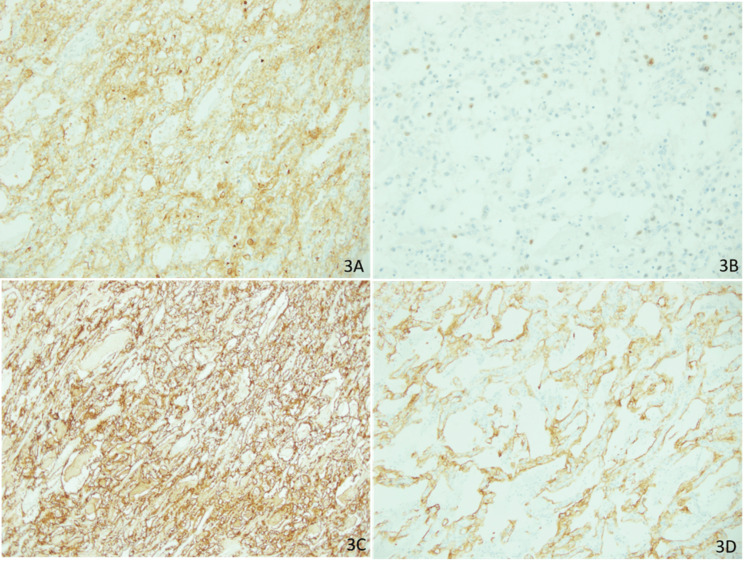
Immunohistochemistry 3A: CD10-positive; 3B: PAX8 patchy nuclear staining; 3C: EMA-positive; 3D: CAM5.2-positive

On gross examination of the small bowel resection specimen, a polypoid mass measuring 8 x 5 x 5 cm and exhibiting both exophytic and endophytic growth was seen (Figures 1A, 1B). The mass had penetrated through the bowel wall and reached the mesentery, with clear resection margins. On microscopic examination, the tumor was arranged in a trabecular pattern and composed of cells with clear to faint eosinophilic cytoplasm with well-defined cytoplasmic borders (Figures 2A-2D). Focal suspicious lympho-vascular invasion was appreciated. On immunohistochemistry, the tumor cells were positive for pan-cytokeratin (AE1/AE3, CAM5.2, EMA), PAX8, and CD10 (Figures 3A-3D). The tumor cells were immunonegative for CK7 and CK20. These findings were consistent with metastatic clear cell RCC extending from mucosa to serosa, with clear margins (stage IV).

## Discussion

Metastasis to distant sites is a common feature of RCC, with 30% of patients being diagnosed with metastasis at the time of presentation of the primary tumor. The small bowel is the least common site of metastasis from RCC, with an incidence of just 0.7% [1]. There is a paucity of evidence in the literature, with limited reports of RCC metastasis to the ileum, especially following nephrectomy, as in our case. Studies have highlighted the hematogenous route of spread, especially to the lung, which is the most common organ for metastasis followed by metastasis elsewhere [7]. However, recent studies have shed light on other routes and shown that lymphatic, trans-coelomic, and direct invasion can precede hematogenous spread.

The likelihood of RCC recurring after 10 years is relatively low but not impossible. Our patient underwent a radical nephrectomy 10 years prior. Recurrence has been reported after post-curative nephrectomy from eight months to up to 20 years. Most cases were treated with tumor resection and end-to-end anastomosis, while supportive care and palliation were provided in cases of late-stage metastasis with oligometastases [8-18].

Our review of the literature indicates that the recent increase in the number of case reports is due to an increased incidence of metastasis to the small bowel over the last two decades. This is validated by the increase in targeted therapies used to treat RCC [1]. Over the last 20 years, research into the pathophysiology of mRCC has enabled the shift from high-dose interleukin-2 and interferon-α to more targeted and efficacious therapies. Targeted therapies that are currently available involve vascular endothelial growth factor receptor (VEGF-R) inhibitors and chemotherapy with checkpoint inhibitors. This development in therapy has led to an increase in the median survival rate of patients with RCC, potentially allowing enough time for RCC to metastasize to places such as the small bowel and become symptomatic [1].

Immunohistochemical (IHC) analyses of biopsies in the case reports align with RCC in all cases. Some reports have mentioned specific IHC markers. The common findings include CD10(+), CK7(-), CK20(-), membrane positivity for vimentin, EMA, and pan-cytokeratin. This is in line with the IHC results in the biopsy of this case, which confirmed that the tumor was an RCC [15-19].

## Conclusions

Our case report contributes to the growing body of evidence on RCC metastasis to the small bowel, highlighting the need for conducting more retrospective cohort studies to determine its true prevalence. Small bowel metastasis should be considered in patients presenting with melena, abdominal pain, nausea, and a history of RCC, with or without nephrectomy. While rare, metastasis of RCC to the small bowel should not be overlooked. Vigilant monitoring and follow-up care are essential due to the varying time frame of recurrence and metastasis. Early detection and proactive management are critical for optimal outcomes in these patients.
